# Cross-Talk and Multiple Control of Target of Rapamycin (TOR) in Sclerotinia sclerotiorum

**DOI:** 10.1128/spectrum.00013-23

**Published:** 2023-03-21

**Authors:** Wenli Jiao, Weichen Ding, Jeffrey A. Rollins, Jinliang Liu, Yanhua Zhang, Xianghui Zhang, Hongyu Pan

**Affiliations:** a College of Plant Sciences, Jilin University, Changchun, China; b Department of Plant Pathology, University of Florida, Gainesville, Florida, USA; Institute of Biotechnology

**Keywords:** *Sclerotinia sclerotiorum*, TOR, MAPK, autophagy, pathogenicity, mitogen-activated protein kinases

## Abstract

Sclerotinia sclerotiorum is a necrotrophic phytopathogenic fungus that cross-talks with its hosts for control of cell-death pathways for colonization. Target of rapamycin (TOR) is a central regulator that controls cell growth, intracellular metabolism, and stress responses in a variety of eukaryotes, but little is known about TOR signaling in *S. sclerotiorum*. In this study, we identified a conserved TOR signaling pathway and characterized SsTOR as a critical component of this pathway. Hyphal growth of *S. sclerotiorum* was retarded by silencing SsTOR, moreover, sclerotia and compound appressoria formation were severely disrupted. Notably, pathogenicity assays of strains shows that the virulence of the SsTOR-silenced strains were dramatically decreased. SsTOR was determined to participate in cell wall integrity (CWI) by regulating the phosphorylation level of SsSmk3, a core MAP kinase in the CWI pathway. Importantly, the inactivation of SsTOR induced autophagy in *S. sclerotiorum* potentially through SsAtg1 and SsAtg13. Taken together, our results suggest that SsTOR is a global regulator controlling cell growth, stress responses, cell wall integrity, autophagy, and virulence of *S. sclerotiorum*.

**IMPORTANCE** TOR is a conserved protein kinase that regulates cell growth and metabolism in response to growth factors and nutrient abundance. Here, we used gene silencing to characterize SsTOR, which is a critical component of TOR signaling pathway. SsTOR-silenced strains have limited mycelium growth, and the virulence of the SsTOR-silenced strains was decreased. Phosphorylation analysis indicated that SsTOR influenced CWI by regulating the phosphorylation level of SsSmk3. Autophagy is essential to preserve cellular homeostasis in response to cellular and environmental stresses. Inactivation of SsTOR induced autophagy in *S. sclerotiorum* potentially through SsAtg1 and SsAtg13. These findings further indicated that SsTOR is a global regulator of the growth, development, and pathogenicity of *S. sclerotiorum* in multiple ways.

## INTRODUCTION

Sclerotinia sclerotiorum is a damaging plant pathogen of worldwide distribution that causes blighting and stem and crown rot of numerous crops (1–4). Sclerotia, which are soilborne, function as the primary source of infection either directly or through their production of ascospores. Sclerotia development is controlled by MAPK, cAMP, autophagy and other signaling pathways (5–9). The components of different signal transduction pathways, however, and how the different pathways cross talk, is not completely understood.

Target of rapamycin (TOR), an evolutionarily conserved Ser/Thr protein kinase, is the indispensable regulator controlling intracellular metabolism and cell growth by activating a battery of biosynthetic and metabolic pathways, including transcription and ribosome biogenesis, protein synthesis, mRNA degradation, and autophagy (10, 11). The TOR kinases are assembled into two multisubunit complexes, namely, TORC1 (TOR complex1) and TORC2 (12). Saccharomyces cerevisiae has two TOR proteins termed TOR1 and TOR2, which integrate into two distinct TOR complexes (TORC1 and TORC2) (13). TORC1 contains either TOR1 or TOR2, KOG1, TCO89, and LST8, which participates in the synthesis of protein and ribosome, autophagy pathway, and controls cell growth process in response to environmental signals (14), whereas TORC2 possesses TOR2, LST8, BIT61, AVO1, AVO2, and AVO3 and regulates spatial features of cell growth such as the cytoskeleton and polarity (12, 15, 16). The similar composition of TORCs in widely divergent kingdoms such as metazoans and fungi suggested these complexes are broadly conserved among all eukaryotes (10). Thus far, despite functional importance, the contribution of the TOR signaling pathway in regulating sclerotia formation and virulence in *S. sclerotiorum* remains uncharacterized.

Interestingly, in filamentous fungi such as Magnaporthe oryzae, Fusarium graminearum, Neurospora crassa, and Aspergillus species only a single TOR kinase is present (17). However, the function of TOR kinase in plant-pathogenic fungi and the genetic pathways associated with TOR are largely uncharacterized (18). Rapamycin, a TOR activity inhibitor, is effective against F. graminearum, Verticillium dahliae (19), and Botrytis cinerea (20). In F. graminearum, FgTap42 is a key downstream factor of FgTOR and interacts with FgPp2A and FgPpg1 to regulate vegetative differentiation and pathogenicity (21). TOR regulates mycelial growth and spore production and affects the expression of cell wall degrading enzymes (CWDEs) in *V. dahlia* (19). Target of rapamycin controls hyphal growth and pathogenicity through *FoTIP4* in Fusarium oxysporum (11). However, questions remain unanswered on how this pathway regulates and impacts various cellular processes, including virulence in necrotrophic plant-pathogenic fungi.

In a preliminary study, we found that rapamycin is a very strong inhibitor of *S. sclerotiorum* growth and sclerotia formation. In this study, we found that the mycelial growth of *S. sclerotiorum* was retarded by silencing SsTOR expression; moreover, sclerotia and compound appressoria formation were severely disrupted. SsTOR plays important roles in cell wall integrity by regulating the phosphorylation of SsSmk3, a key MAP kinase in the cell wall integrity pathway. Importantly, the inactivation of SsTOR induced autophagy potentially through SsAtg1 and SsAtg13. Thus, SsTOR is a central regulator that controls cell growth, stress responses, cell wall integrity, autophagy, and virulence of *S. sclerotiorum*.

## RESULTS

### Sequence analysis of SsTOR.

**FIG 1:**
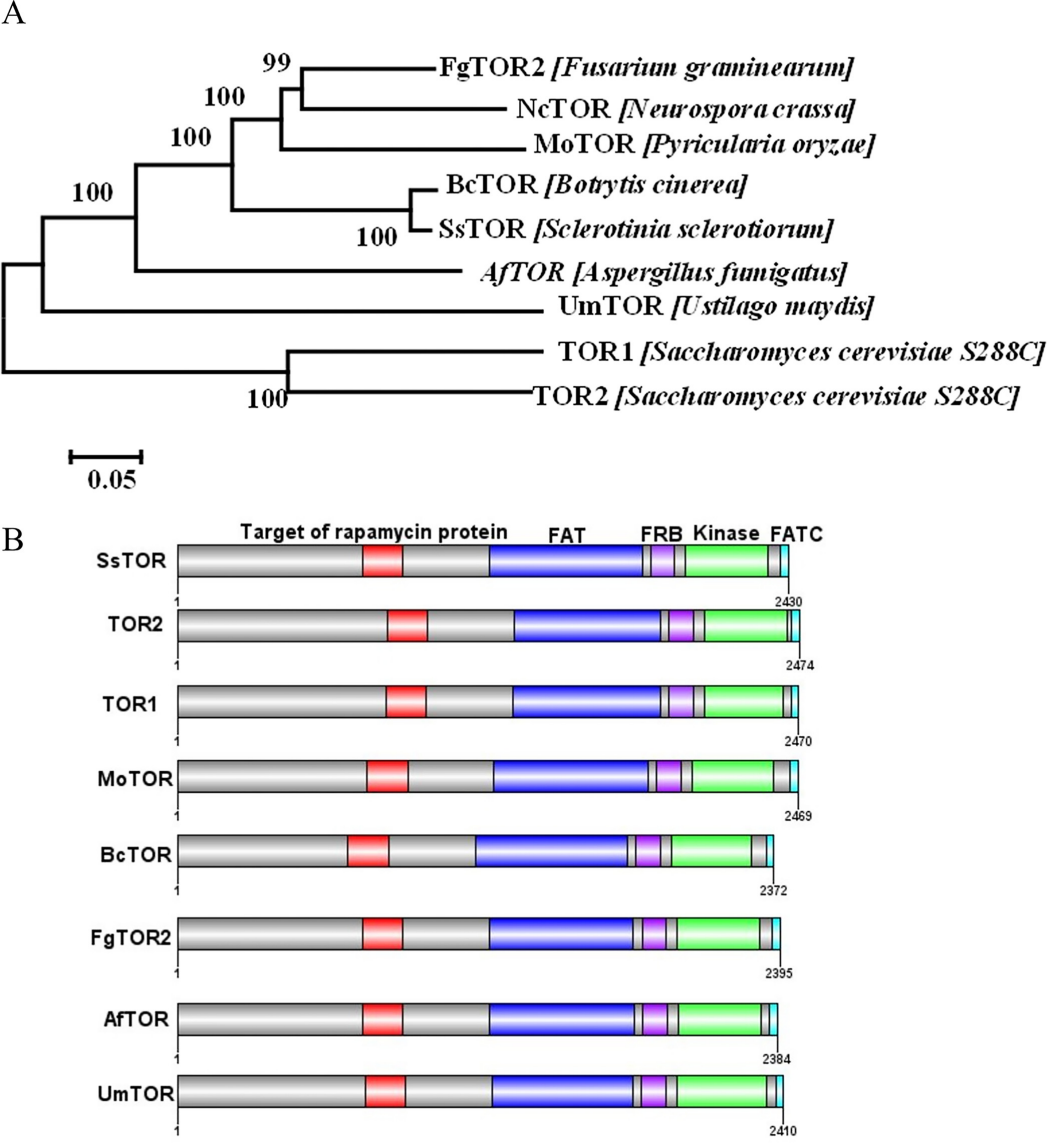
Sequence analysis of SsTOR. (A) Dendrogram of SsTOR. Mega 7.0 was used to construct the phylogenetic tree. Sequences are provided in Table S1. (B) Domain conservation and organization for SsTOR and homologs. FAT (dark blue): Phosphatidylinositol kinase (PIK)-related kinases ~550-amino acid-long FAT (FRAP, ATM, TRRAP) domain, FRB (purple): FKBP12-rapamycin binding domain superfamily, Phosphatidylinositol 3-/4-kinase (green) and FRAP-ATM-TRRAP-C-terminal (FATC) domain (light blue). Conserved domain information was analyzed by interpro (http://www.ebi.ac.uk/interpro) and the distribution of domains was visualization by GPS 2.0.

The TOR signaling pathway has not been characterized in *S. sclerotiorum*; therefore, BLASTp analysis of the *S. sclerotiorum* genome database was performed using sequences of the TOR signaling pathway components of Saccharomyces cerevisiae and *Saccharomyces pombe*. TOR kinases (TOR1 and TOR2) are central to TORC1 and TORC2 in most eukaryotes such as S. cerevisiae and S. pombe. In contrast, in *S. sclerotiorum*, there is a single TOR kinase homolog (Sscle_02g011660). TORC1 and TORC2 perceive different signals and contain diverse accessory proteins. TORC1 contains the RAPTOR (Regulatory Associated Protein of TOR) protein, while TORC2 contains two specific proteins called RICTOR (Rapamycin Insensitive Companion of TOR) and SIN1 (Stress-activated protein kinase I interacting protein 1). Interestingly, in the *S. sclerotiorum* genome, all these putative homologues encoding specific components of both complexes are encoded (Table 1). In order to determine whether SsTOR was evolutionarily conserved we made a comparison of homologous proteins from the plant-pathogenic fungi Magnaporthe oryzae (XP_003710485.1), Botrytis cinerea (XP_024546753.1), and F. graminearum (XP_011320682.1) (Fig. 1A). Subsequently, we used InterPro to analyze SsTOR and its homologous sequences and found that TOR protein is highly conserved in necrotrophic and biotrophic fungi, and all contain FAT (dark blue), FRB (purple), Phosphatidylinositol 3-/4-kinase (green), and FATC domains (light blue) with conserved intraprotein organization (Fig. 1B).

**TABLE 1:** Putative components of the TOR signaling pathway in *S. sclerotiorum*

Protein name	Saccharomyces cerevisiae *S288C* (E value, per.Ident)	*Saccharomyces pombe* (E value, per.Ident)	*S. sclerotiorum*	Annotation no.
Target of rapamycin (TOR)	Tor1 (0.0, 47.25%) Tor2 (0.0, 48.7%)	Tor1 (0.0, 47.45%) Tor2 (0.0, 53.22%)	SsTOR	sscle_02g011660
Lethal with SEC- 13 protein 8 (LST8)	Lst8 (7e^−154^, 61.78%)	Wat1 (1e^−157^, 65.08%)	SsLst8	sscle_09g069520
FK506 binding protein 12 (FKBP12)	FPR1 (5e^−33^, 57.66%)	Fkh1 (4e^−41^, 59.32%)	SsFKBP12	sscle_10g075850
Regulatory associate protein of TOR (RAPTOR)	Kog1 (1e^−136^, 54.65%)	Mip1 (0.0, 47.47%)	SsKog1	sscle_15g105080
Rapamycin- insensitive companion of TOR (RICTOR)	Avo3/TSC11 (1e^−131^, 29.51%)	Ste20 (0.0, 37.20%)	SsAvo3	sscle_03g029640
Adhere voraciously to TOR2	Avo2 (2e^−10^, 33.61%)	-	SsAvo2	sscle_16g110420
Stress- activated MAP kinase- interacting protein 1 (SIN1)	Avo1 (5e^−18^, 36.75%)	Sin1 (5e^−58^, 31.24%)	SsAvo1	sscle_14g098330
AGC family protein kinase	Ypk1 (3^e-177^, 46.45%) Ypk2 (1e^−176^, 48.00%)	Gad8 (0.0, 51.21%)	SsGad8	sscle_11g085600
Ribosomal protein S6 kinase (S6K)	Sch9 (0.0, 52.89%)	Sck1 (0.0, 51.72%) Sck2 (2e^−179^, 54.32%)	SsSch9	sscle_01g011240
Rag GTPase GTR1	Gtr1 (3e^−88^, 50.79%)	Gtr1 (5e^−111^, 52.22%)	SsGtr1	sscle_01g004410
GTP-binding protein gtr2	Gtr2 (9e^−88^,42.43%)	Gtr2 (2e^−128^, 58.70%)	SsGtr2	sscle_06g053500
Rheb GTPase Rhb1	Rhb1 (2e^−99^, 72.83%)	Rhb1 (2e^−99^, 72.83%)	SsRhb1	sscle_03g029100

### Silence of SsTOR results in abnormal hyphae growth and sclerotia formation.

**FIG 2:**
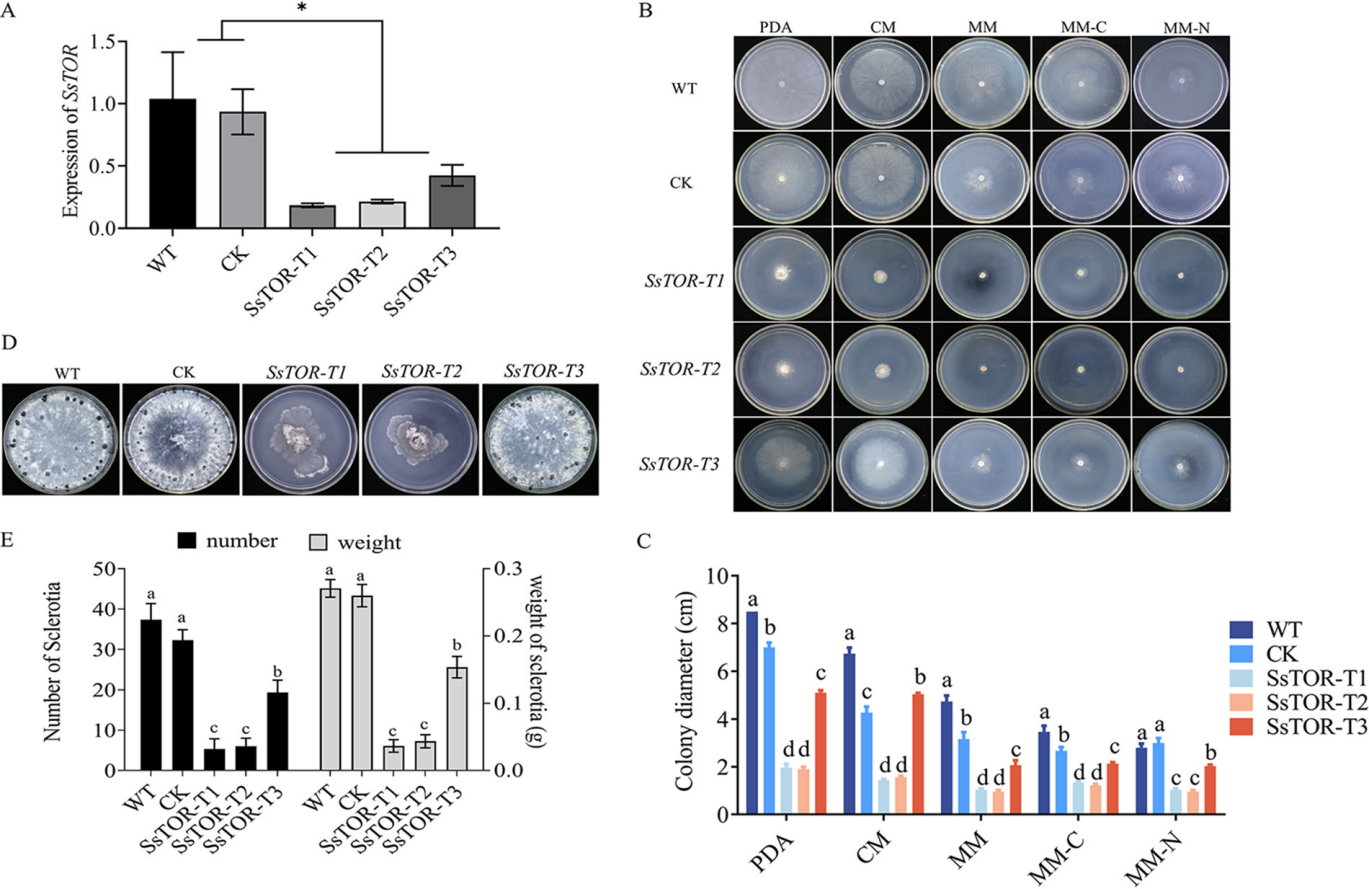
Silence of SsTOR results in abnormal hyphae growth and sclerotia formation. (A) Relative expression of *SsTOR* in WT, CK and silenced strains. (B) Vegetative growth of *SsTOR*-T1, *SsTOR*-T2 and *SsTOR*-T3. The colony diameters were measured after 2d incubation (C), letter means the significant differences between the WT and indicated mutants. Sclerotia formation of *SsTOR*-T1, *SsTOR*-T2 and *SsTOR*-T3 (D, E). The Error bars represent the standard deviations (SDs) from three independent experiments and the analysis of significant differences between the WT and indicated mutants was performed by *t* test.

S. cerevisiae has two TOR kinase genes; however, *in silico* analysis indicated that SsTOR is the only TOR kinase gene predicted in *S. sclerotiorum*. To further determine the function of SsTOR, we experimented with creating a null mutant but were unsuccessful, proposing that SsTOR was essential in *S. sclerotiorum.* Therefore, we used p-Slient 1 vector to construct a silencing vector for RNAi of SsTOR and measured the expression of silencing transformants by qPCR. Three silencing strains with different SsTOR silencing efficiencies were obtained: *SsTOR*-T1, *SsTOR*-T2, and *SsTOR*-T3 (Fig. 2A). TOR1 deletion has no significant effect on the growth and development of S. cerevisiae, on the contrary, loss of TOR2 is lethal.

We evaluated the vegetative growth of the *SsTOR*-T1, *SsTOR*-T2, and *SsTOR*-T3 on growth medium, including PDA, CM, MM, MM-C and MM-N. *SsTOR*-T1, *SsTOR*-T2, and *SsTOR*-T3 all showed reduced radial growth. Additionally, under the condition of nutrient restriction, mycelia growth was severely limited (Fig. 2B and C). After 4 weeks of culture, the sclerotia of *SsTOR*-T1, *SsTOR*-T2, and *SsTOR*-T3 developed abnormally and the number and weight of sclerotia was decreased significantly (Fig. 2d and e). These results indicate that TOR signaling pathway plays an important role in the growth and development of sclerotia in *S. sclerotiorum*.

### SsTOR is involved in abiotic stress response in *S. sclerotiorum*.

**FIG 3:**
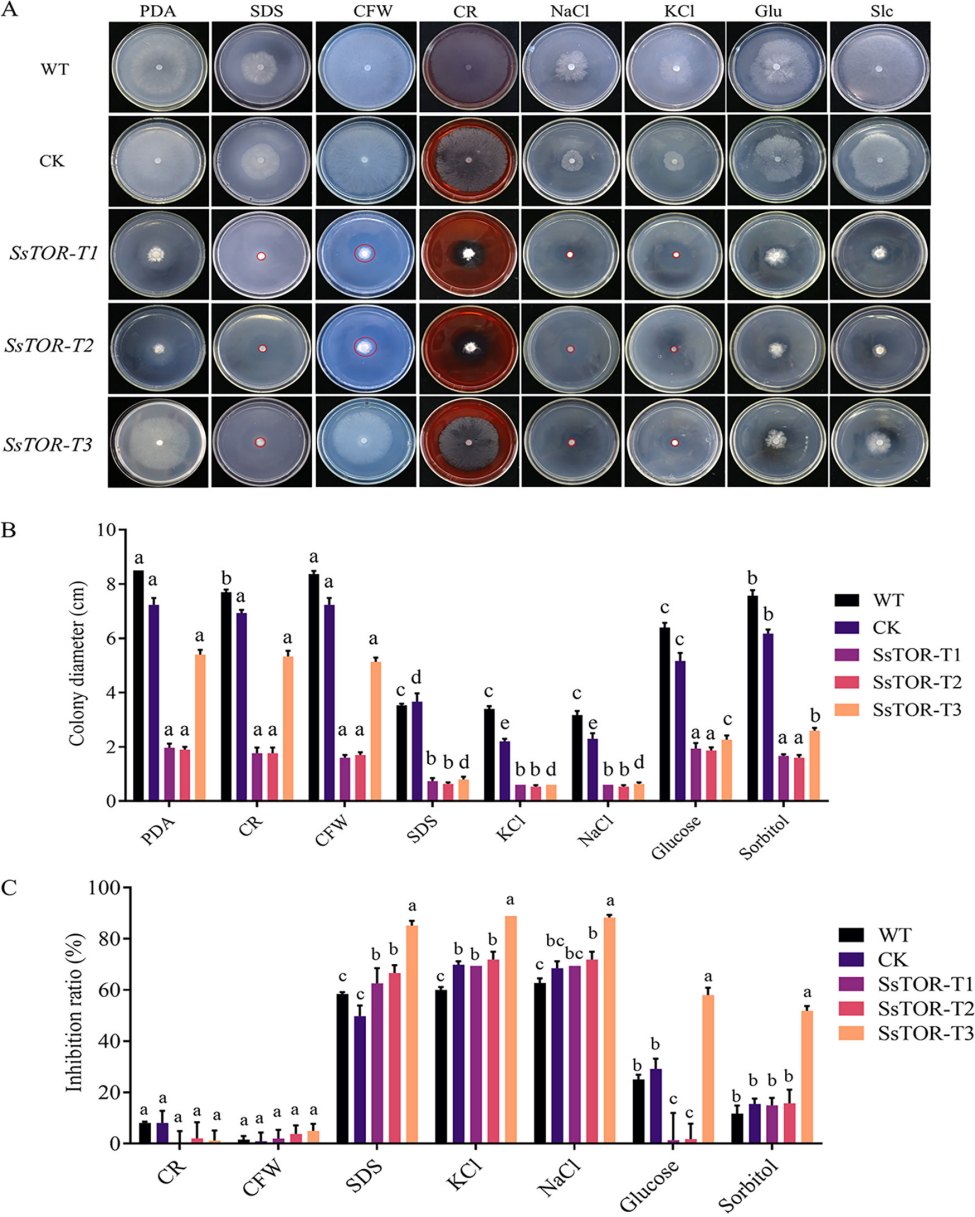
SsTOR is involved in abiotic stress response in *S. sclerotiorum*. (A) Growth of WT, CK and SsTOR silenced strains *SsTOR*-T1, *SsTOR*-T2 and *SsTOR*-T3 on different abiotic stress. The colony diameters were measured after 2d incubation (B), the inhibition rates = (colony diameter of strain without stress—colony diameter of strain with stress)/colony diameter of strain without stress ×100% (C). The Error bars represent the standard deviations (SDs) from three independent experiments and the analysis of significant difference between the WT and indicated mutants by *t* test.

In order to explore the response of the TOR signaling pathway to different stresses, *S. sclerotiorum* was treated with various cell wall inhibitors and osmotic stresses. When exogenous cell wall inhibitors Congo red (CR) and calcofluor white (CFW) were added, SsTOR-silenced strains *SsTOR*-T1, *SsTOR*-T2 and *SsTOR*-T3 showed high tolerance relative to the untreated growth control (Fig. 3A and B). When 1 M NaCl and KCl were added externally, colony diameters of SsTOR silenced strains *SsTOR*-T1, *SsTOR*-T2. and *SsTOR-*T3 were significantly reduced, and the inhibition rate was up to 88%, which was significantly higher than that of the wild type. However, in the medium supplemented with 1 M glucose and sorbitol, the colony diameter of *SsTOR*-T1 and *SsTOR*-T2 was not significantly different from that of PDA medium, and the growth inhibition rate was significantly lower than that of wild-type and CK, but the phenotype of *SsTOR-*T3 was different from that of *SsTOR-*T1 and *SsTOR-*T2, probably because of the various silencing efficiency (Fig. 3).

### TOR signaling affects cell wall integrity of *S. sclerotiorum* through altered SsSmk3 phosphorylation.

**FIG 4:**
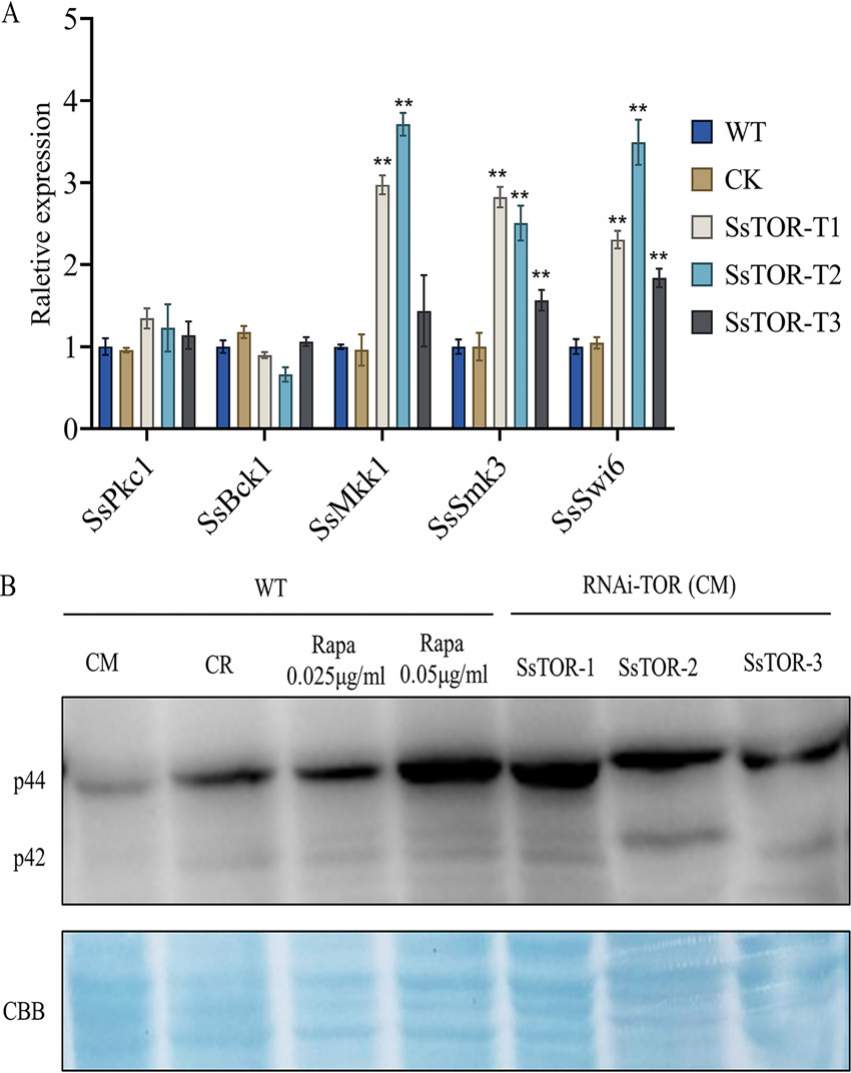
TOR signaling affects the cell wall integrity signaling pathway of *S. sclerotiorum* through altered SsSmk3 phosphorylation. (A) Relative expression of key genes in the CWI-MAPK pathway. Hyphae were collected from cellophane-overlaid media 2d postinoculation. The data are presented as the mean ± SD of *n* = 3 independent experiments. Asterisks represent significant difference between the WT and indicated mutants (*, *P* < 0.05 by *t* test). (B) Inactivation of SsTOR increased phosphorylation levels of SsSmk3. Mycelia were cultured in CM liquid for 36h, and then treated with Congo red and rapamycin for 6h. Total proteins were separated on 12% SDS-PAGE, stained with Coomassie brilliant blue (CBB) and immunoblotted with phospho-p44/42 MAP kinase antibody (Cell Signaling Technology, Beverly, MA, USA).

The cell wall plays a crucial role in maintaining cell morphology and progression through the cell cycle, resulting in normal growth and development of *S. sclerotiorum*. In order to further determine the influence of the TOR pathway on cell wall synthesis, we analyzed the expression levels of key genes in CWI-MAPK pathway, and found that *SsMkk1* (*Sscle_03g029980*), *SsSmk3* (Slt2, *Sscle_08g066770*), and *SsSwi6* (*Sscle_10g075380*) showed upregulated expression in the silenced SsTOR strains (Fig. 4A).

Slt2 is a key kinase within the cell wall integrity pathway. In order to explore the correlation between the TOR signaling pathway and the SLT2-MAPK pathway, we determined phosphorylation levels of Slt2 homolog SsSmk3 in WT and SsTOR-silenced strains *SsTOR*-T1, *SsTOR*-T2, and *SsTOR*-T3 under treatment with the cell wall inhibitor Congo red (CR) and the TOR inhibitor rapamycin. Western blot results showed that after 6h treatment with CR and rapamycin, the phosphorylation level of SsSmk3 in WT was significantly higher than that in untreated complete medium (CM). Similarly, the phosphorylation level of SsSmk3 in untreated *SsTOR-s*ilenced strains *SsTOR-T*1, *SsTOR-T*2, and *SsTOR*-T3 was increased (Fig. 4B). These results suggested that SsTOR kinase affects the cell wall integrity pathway of *S. sclerotiorum* by regulating the phosphorylation level of SsSmk3.

### SsTOR is necessary for proper compound appressoria development and pathogenicity.

**FIG 5:**
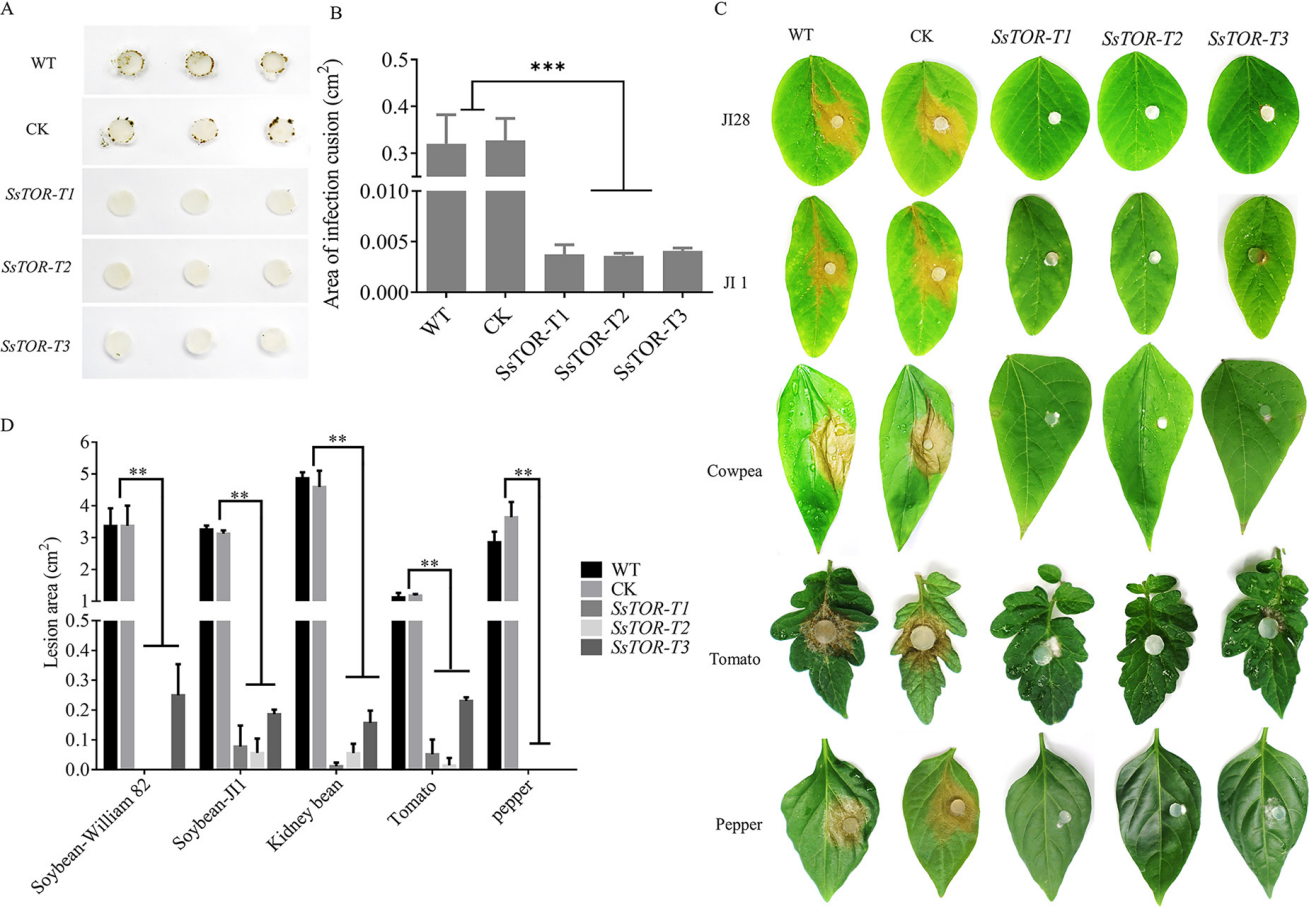
SsTOR is necessary for compound appressoria formation and pathogenicity. (A, B) Effect of SsTOR-silencing strains on compound appressoria formation. Image J was used to analysis of compound appressoria. *, *P* < 0.05 compared with WT and CK (*t* test). (C, D) The virulence of SsTOR- silencing strains *SsTOR*-T1, *SsTOR*-T2 and *SsTOR*-T3 was significantly reduced. Lesion area were calculated by image J. The data are presented as the mean ± SD of *n* = 3 independent experiments.

The compound appressoria produced by *SsTOR*-T1, *SsTOR*-T2 and *SsTOR*-T3 mutants were abnormal and scarcely produced, which was significantly different from the wild-type (Fig. 5A and B). The compound appressoria are the key structure for the direct infection of plant tissues by *S. sclerotiorum*, subsequently, we analyzed the pathogenicity of *SsTOR*-T1, *SsTOR*-T2, and *SsTOR*-T3 mutants The virulence of *SsTOR*-T1-, *SsTOR*-T2-, and *SsTOR*-T3-silenced strains were significantly lower than that of the wild type (Fig. 5C and D). After 48h inoculation, the wild-type could form obvious water-stained pale brown lesion on the leaves of different hosts with blighting necrosis. However, the virulence of *SsTOR*-T1-, *SsTOR*-T2-, and *SsTOR*-T3-silenced strains was significantly lower than that of the wild type. There was no obvious symptom development around the agar disk for most hosts and strains and no lesion expansion was observed on any of the hosts, which was consistent with the phenotype of compound appressoria formation defect. Results indicated that the effect of *SsTOR* on the pathogenicity of *S. sclerotiorum* is multifactorial.

### The inactivation of SsTOR induces autophagy.

Under nutrient limitation, TOR inactivation induces autophagy, a catabolic membrane-trafficking process within the cell. During this process, a large number of cytosolic components are nonselectively enclosed within a double-membrane and delivered to the vacuole for degradation to recycle needed nutrients or degrade toxic components. Accordingly, we investigated whether TOR signaling inactivation induces autophagy.

**FIG 6:**
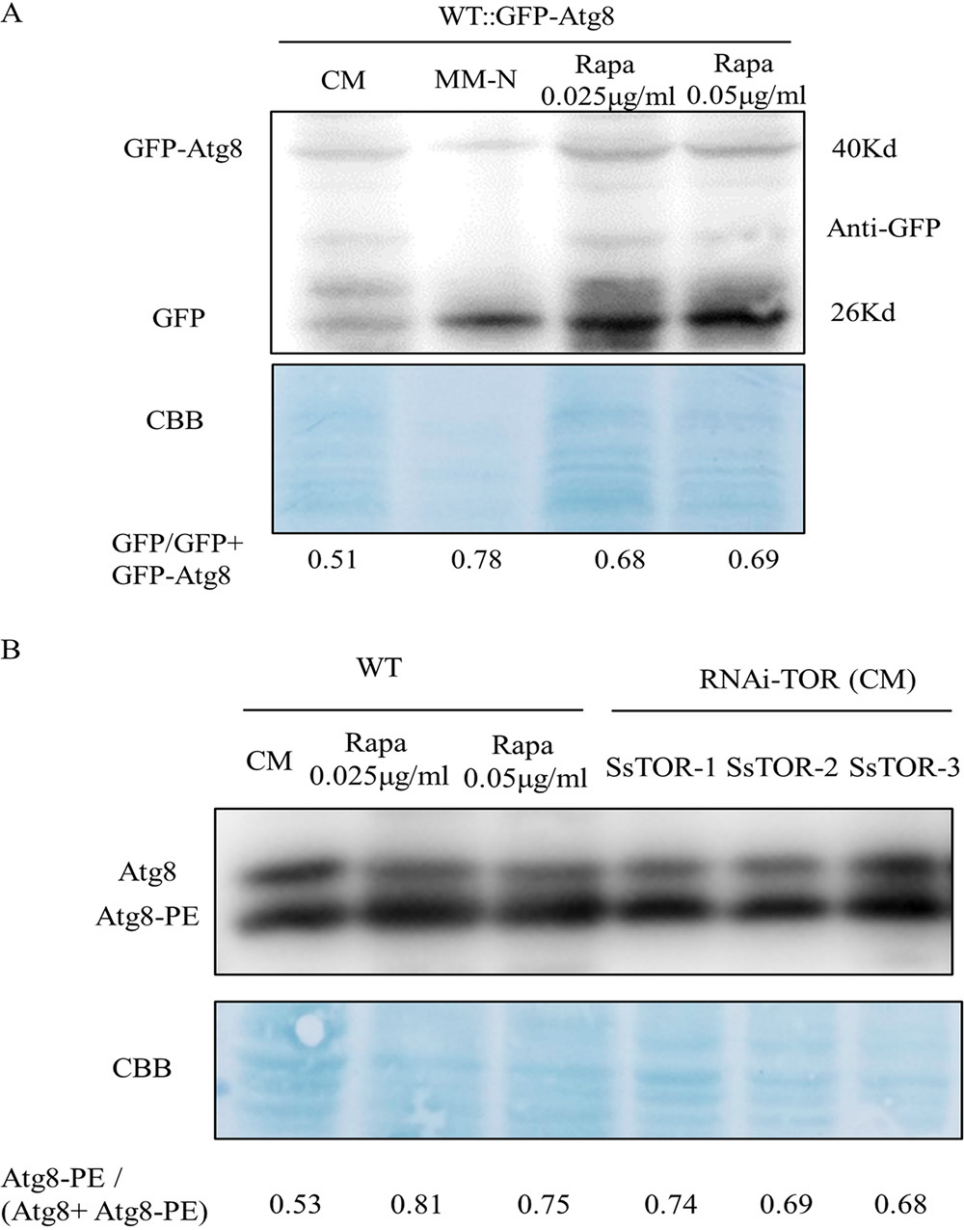
The inactivation of SsTOR induces autophagy. (A) Rapamycin-induced autophagy in *S. sclerotiroum*. Hypha cultivate in liquid CM 36h, then transfer to CM containing rapamycin and MM-N. Total protein were extracted after 6 h of incubation. Anti-GFP was used to measuring proteolyzed of SsAtg8 under starvation and rapamycin. Image J was used to measure the gray value and the autophagy efficiency was calculated by the Atg8-PE/Atg8+Atg8-PE. (B) SsTOR-silenced strains induced autophagy. Anti-Atg8 (Filamentous fungi) pAb was used to detected autophagy.

We added different concentrations of rapamycin to a GFP-tagged SsAtg8 (an autophagy marker) strain in the wild-type background to inhibit TOR activity. We found that when rapamycin inhibited TOR activity, the signal from the autophagy marker increased (Fig. 6A). Subsequently, ATG8 antibody was used to analyze autophagy levels of WT and SsTOR silenced strains. We found that the ATG8-PE content of SsTOR-silenced strains was significantly higher than that of CM liquid culture, similar to that of autophagy induced by exogenous rapamycin (Fig. 6B). The results further suggest that the TOR kinase negatively regulates autophagy. Inactivation of TOR function by rapamycin induces autophagy even in rich nutrient conditions indicating that SsTOR inhibits autophagy.

### SsAtg13 affects *S. sclerotiorum* nutrient utilization.

**FIG 7:**
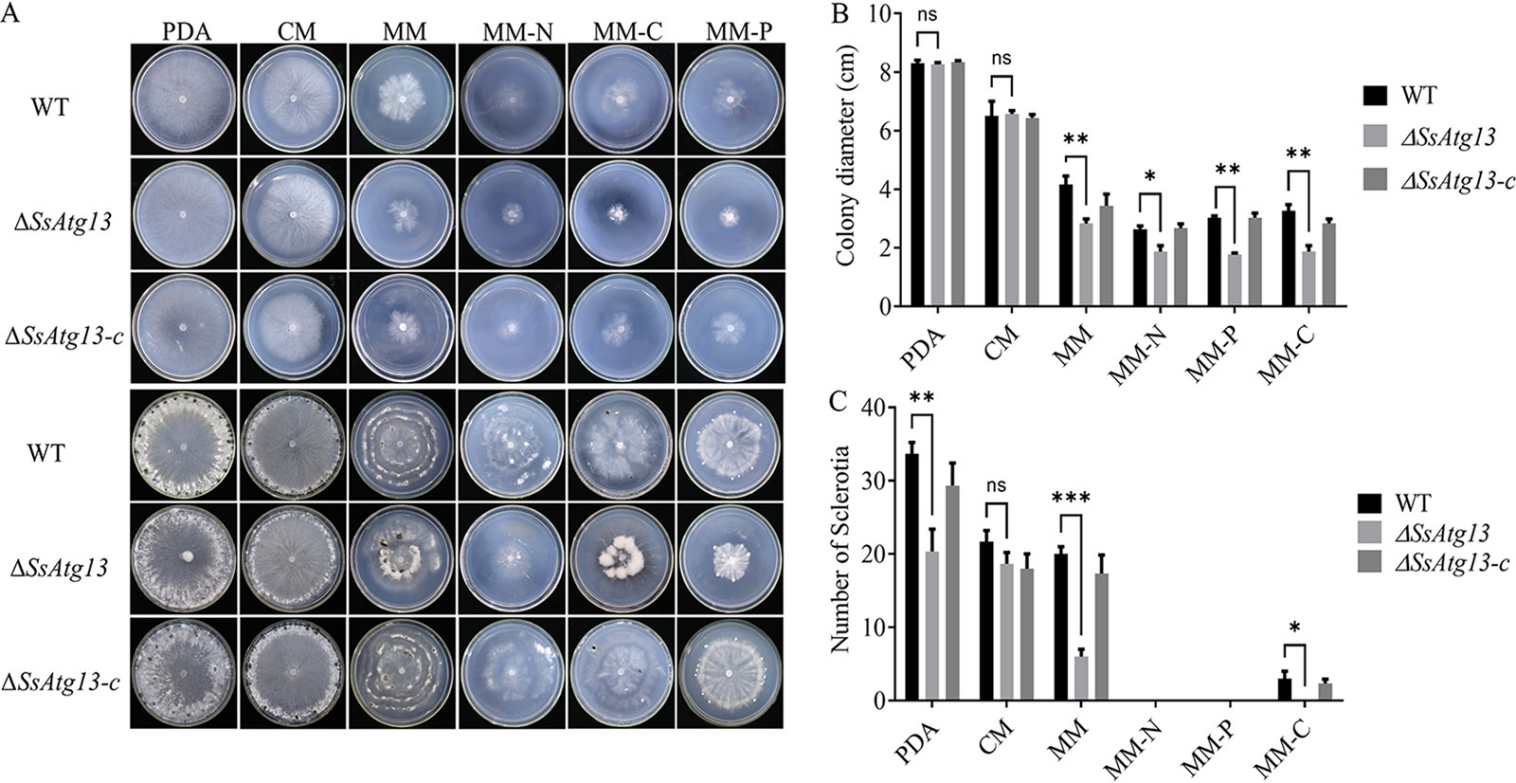
SsAtg13 affects *S. sclerotiorum* nutrient utilization. (A) Colony morphology of strains under different nutrient culture conditions. Colony morphology was photographed at 2d and 14d, respectively. (B) Colony diameters were measured after 2d incubation. (C) Sclerotia were counted after 14d incubation. Error bars mean standard deviation between 3 repetitions. (*, *P* < 0.05).

TOR inactivation induces autophagy primarily at the level of autophagosome formation by negatively regulating the association between the Ser/Thr protein kinase Atg1 with Atg13, a regulatory subunit of Atg1. SsAtg1 is a key kinase in autophagy induction. Therefore, to explore whether TOR inactivation induces autophagy through SsAtg1 (Sscle_12g087380) and SsAtg13 (Sscle_15g103100), we performed gene knockout on SsAtg13 of the SsAtg1 complex, by PCR verification that SsAtg13 was knockout and hygromycin gene showed correct insertion (Fig. S1) and found that nutrient deficiency significantly inhibited the growth of Δ*SsAtg13* (Fig. 7A and B).

### SsAtg1 and SsAtg13 are involved in the autophagy pathway induced by TOR inhibition.

**FIG 8:**
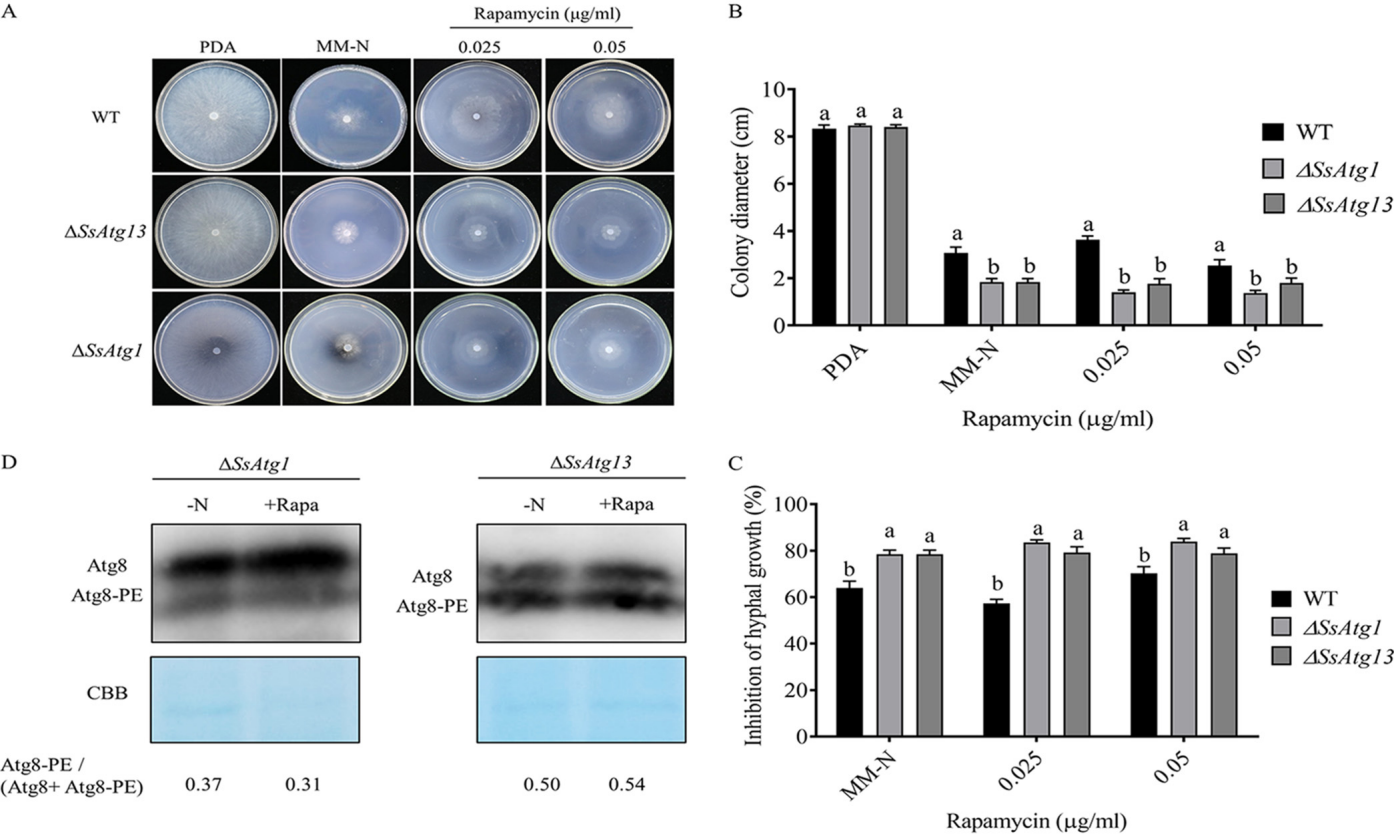
SsAtg1 and SsAtg13 are involved in the autophagy pathway induced by TOR inhibition. (A) Low concentrations of rapamycin significantly inhibited the growth of Δ*SsAtg1* and Δ*SsAtg13* mutants. (B) Colony diameters were measured after 2d of incubation. (C) inhibition rates = (colony diameter of strain without stress–colony diameter of strain with stress)/colony diameter of strain without stress ×100%. (D) Rapamycin treatment and nitrogen starvation effect on ATG8 accumulation was examined in Δ*SsAtg1* and Δ*SsAtg13* mutants.

Treatment with low concentrations of rapamycin significantly inhibited the growth of Δ*SsAtg1* and Δ*SsAtg13* loss-of-function mutants (Fig. 8A and B, C). Autophagy in Δ*SsAtg1* and Δ*SsAtg13* was measured by following the levels of Atg8 with an anti-Atg8 antibody. This assay found that the exogenous addition of rapamycin did not produce an increase of Atg8 levels in Δ*SsAtg1* and Δ*SsAtg13* mutants after rapamycin treatment (Fig. 8D). These results suggest that SsAtg1 and SsAtg13 are involved in the autophagy pathway induced by TOR inhibition.

## DISCUSSION

TOR is an evolutionarily conserved protein kinase that regulates cell growth and metabolism in response to growth factors, hormones, cellular energy status, and nutrient abundance (12, 14, 21, 22). In this study, we provide some insights into how SsTOR modulates cell growth and pathogenicity through control of multiple cellular processes in *S. sclerotiorum*. We identified the core component of the TOR signaling pathway, SsTOR, which has high similarity with the kinase domain of TOR proteins in other organisms, however, different from S. cerevisiae, there is only one TOR kinase gene predicted in *S. sclerotiorum*. Functional studies of SsTOR in *S. sclerotiorum* suggested that SsTOR was lethal. After gene silencing, silenced transformants exhibited effects on mycelial growth and sclerotia formation.

The fungal cell wall is an external rigid structure composed of polysaccharides and glycoproteins. It gives shape and integrity to the cell and is continuously remodeled during growth and development. The composition and thickness of the cell wall changes when cells encounter different environmental stresses and, the cell wall integrity (CWI) pathway is tightly regulated in response to different signaling pathways (23, 24). The CWI pathway is activated through Slg1/Wsc1, Wsc2, Wsc3, Mid2, and Mtl1, which can couple with Rho1 and activate the MAPK cascade (Pkc, Bck1, Mkk1, and Slt2) (25–27). *SsTOR*-silenced strains are not sensitive to cell wall inhibitors and the mycelium is denser than the wild type. Subsequently, we measured the expression levels of key genes in the CWI pathway and found that the expressions of SsMkk1 and SsSmk3 genes were upregulated. Analysis of the phosphorylation level of SsSmk3 showed that the phosphorylation level of SsSmk3 increased significantly after the inhibition of TOR activity, indicating that cross talk exists between the TOR pathway and MAPK pathway, but its mechanism still needs to be further explored.

Autophagy, an evolutionarily conserved pathway among eukaryotes, is essential to preserve cellular homeostasis in response to cellular and environmental stresses (28, 29). Autophagy is also crucial for a multitude of physiological processes in plant-pathogenic fungi, such as cell proliferation, differentiation, and pathogenicity (30–34). In yeast, inhibition of TOR activity can induce autophagy (10). In our study, it was found that autophagy was detected in TOR gene-silenced strains without the application of autophagy inducing conditions in CM medium. This finding is consistent with the effects seen with the exogenous addition of the TOR inhibitor rapamycin and MM-N treatment of WT indicating that TOR is involved in the autophagy pathway in *S. sclerotiorum*. Control of autophagy by TOR occurs primarily at the induction step and involves activation of the Atg1 kinase, a conserved component of the autophagic machinery. Subsequently, we determined the relationship between TOR inhibition-induced autophagy and SsAtg1 and SsAtg13 using Δ*SsAtg1* and Δ*SsAtg13* mutants. We found that low concentrations of rapamycin significantly inhibited the growth of Δ*SsAtg1* and Δ*SsAtg13*. In addition, an anti-Atg8 antibody was used as a marker of autophagy in Δ*SsAtg1* and Δ*SsAtg13*, and it was found that exogenous rapamycin did not induce an increase in autophagy with Δ*SsAtg1* and Δ*SsAtg13,* indicating that *SsAtg1* and *SsAtg13* were involved in the autophagy pathway induced by TOR activity inhibition.

Previous studies have found that SsAtg1 affects sclerotia development and pathogenicity of *S. sclerotiorum*, but the function of SsAtg13 and its mechanism of action in autophagy remain unclear. Therefore, we conducted gene function verification of SsAtg13, and the results showed that sclerotia and compound appressoria could be formed in *SsATG13* gene deletion mutants. The results showed that the action of SsAtg13 was different from that of Atg13 in different plant pathogens. The absence of *ATG13* in M. oryzae affected its pathogenicity (35). The mycelium growth, sporulation and pathogenicity of *Fgatg13* mutant in F. graminearum were significantly lower than those of the wild type (36). However, the *Ssatg13* mutant did not affect the pathogenicity and the development of compound appressoria in *S. sclerotiorum* (Fig. S2, S3).

In conclusion, TOR regulates the growth, development and pathogenicity of *S. sclerotiorum* in multiple ways. With TOR silencing, hyphal growth was significantly inhibited and sclerotial developed was abnormal. TOR affects cell wall integrity of *S. sclerotiorum* by regulating the phosphorylation of SsSmk3. In addition, autophagy induced by inhibition of TOR activity is related to SsAtg1 and SsAtg13 activities.

## MATERIALS AND METHODS

### Strains, culture condition, and plant materials.

Sclerotinia sclerotiorum UF-1 was used as the wild-type (WT) strain throughout this study (37). All strains were routinely grown on potato dextrose agar (PDA) at 25°C.

### Identification and sequence information of *SsTOR*.

Phylogenetic analysis was performed in MEGA7 by using the Neighbor-Joining method (1000 bootstrap replicates). NCBI (https://www.ncbi.nlm.nih.gov/) and Interpro (http://www.ebi.ac.uk/interpro) were used to analyze the conserved domains of SsTOR. GPS was used to visualize the distribution of conserved domains.

### Plasmid constructs and transformation.

Split-marker PCR was used to obtain the knockout fragments: primers SsAtg13F1/SsAtg13R1 and SsAtg13F2/SsAtg13R2 were used to generate the SsAtg13 upstream FR1 and downstream FR2. The hygromycin gene cassette was amplified from the pUCATPH vector with primers M13R/NLC37 and M13F/NLC38 to obtain overlapping HY and YG fragments (38, 39). Then, the FR1/HY and FR2/YG amplicons were used as the templates to obtain knockout fragments. pSilent-Dual1 was used for gene silencing, cDNA fragment was amplified with pSD1-Tor1-F1/R1, pSD1-Tor1-F2/R2, and pSD1-Tor1-F3/R3. Plasmid pYF11 was used for the overexpression of genes. The PEG-mediated transformation method was used to transform the two sequences into the protoplasts of *S. sclerotiorum* as previously described (40). Transformants were purified by using Hygromycin (100 μg/mL) and Geneticin (100 μg/mL) selected hyphal-tip at least five times, respectively. Information of primers are in Table s1.

### Stress treatment.

Congo red (CR, 0.5 mg/mL), Calcofluor white (CFW, 20 μg/mL), and 0.01% sodium dodecyl sulfate (SDS) were supplement to PDA as cell wall synthesis inhibitors; 1 M KCl, NaCl, sorbitol and glucose were added to PDA as abiotic stress inducers. CM (0.2g KH_2_PO_4_, 0.25g MgSO_4_·7H_2_O, 0.15 g NaCl, 1g Ca[NO_3_]_2_·4H_2_O, 10g glucose, 1g yeast extract, 1g casein hydrolysate, 15g agar), MM (CM without 1g yeast extract and 1g casein hydrolysate), MM-N (remove Ca[NO_3_]_2_·4H_2_O from MM), MM-C (remove glucose from MM), MM-P (remove KH_2_PO_4_ from MM) were used as different nutrient medium.

### Analysis of compound appressoria, sclerotia formation and pathogenicity.

Compound appressoria of strains were observed by inoculated agar disk (d = 0.7 mm) on hydrophobic surface (glass slides) in a humidified box at 25 °C. Image J was used to calculate the areas of compound appressoria. Sclerotia development was photographed and counted 4 weeks after incubation. Pathogenicity analysis of strains was performed by mycelia-colonized plugs (d = 0.5 mm) inoculated on leaves of soybean (JI 1 and William 82), tomato, pepper, and kidney bean and incubated in a humidity chamber. Lesion areas were measured by image J after 48h inoculation. Experiments were conducted three times.

### Quantitative real-time PCR analysis.

Mycelia of wild-type, *SsTOR*-T1, *SsTOR*-T2, and *SsTOR*-T3 silenced strains were obtained on cellophane-overlaid PDA cultures after 48h inoculation. Total RNA was extracted using the TransZol Up Plus RNA kit (TransGen Biotech, Beijing), and cDNA was obtained by HiFiScript gDNA Removal RT MasterMix (Cwbio). TransStart. Green qPCR SuperMix (TransGen Biotech, Beijing) was used to measured relative expression of genes. Actin was used as internal reference and NCBI primer was used to design primers.

### Protein extraction and monitoring autophagy.

Total fungal proteins were extracted with the Total Protein Extraction kit for Microbes with Thick Walls (invert, YT-O15). The proteome was separated on SDS-PAGE gels, transferred to polyvinylidene fluoride (PVDF) membranes and immunoblotted with corresponding antibodies. WT::GFP-SsAtg8 which contain GFP-SsAtg8 was used to detected the occurrence of autophagy under MM-N growth and rapamycin treatment. In addition, Anti-Atg8 (Filamentous fungi) pAb was used to detect autophagy of strains under different treatments, the proportion of Atg8-PE relative to Atg8 was used to determine the degree of autophagy among different strains.
