# Supplementary material for: Cross-Talk and Multiple Control of Target of Rapamycin (TOR) in Sclerotinia sclerotiorum
Source: Microbiol Spectr. 2023 Mar 21;11(2):e00013-23. doi: 10.1128/spectrum.00013-23 (PMC10100786; doi:10.1128/spectrum.00013-23)
Supplement: Supplemental file 1 — Supplemental material. Download spectrum.00013-23-s0001.pdf, PDF file, 0.5 MB [file spectrum.00013-23-s0001.pdf]

Table S1 Primer information table

Primers	Sequence（5’-3’）
pSD1-Tor1-F1	GCTCTAGA AAATGAATTTGTGCGTGATG
pSD1-Tor1-R1	GCTCTAGA TCTGCTTTGGCAAGGTGT
pSD1-Tor1-F2	GCTCTAGA TTGAAGAGGCATCCAGAC
pSD1-Tor1-R2	GCTCTAGA TTGATAGTACAAATCCCAAG
pSD1-Tor1-F3	GCTCTAGA GTTATCTCCTCCAAGCAA
pSD1-Tor1-R3	GCTCTAGA CTATTAGGAACCCAACCA
SsAtg13F1	CGAGGCTCCGTTCTACTATCA
SsAtg13R1	TCCTGTGTGAAATTGTTATCCGCTGCCCGTCCGACTTTCTTC
SsAtg13F2	GTCGTGACTGGGAAAACCCTGGCGGCCGAGTGCTTCTTTCTG
SsAtg13R2	TACTTTCTTCCCACCTAC
M13R	AGCGGATAACAATTTCA**C**ACAGGA
NLC37	GGATGCCTCCGCTCGAAGTA
M13F	CGCCAGGGTTTTCCCAGTCACGAC
NLC38	CGTTGCAAGACCTGCCTGAA
SsAtg13F3	CTGATCCGATGGGTCTTT
SsAtg13R3	ATACTTGGCGAATGTGCG
pYF11-SsAtg13F	GTAGGAACCCAATCTTCAAA ATGCCACATTATCAAGAGGT
pYF11-SsAtg13R	AGCTCCTCGCCCTTGCTCAC CCTGCCCCAACGGCTACCTC
QSwi6F	GCCCGTTTACCCGTTCTACA
QSwi6R	ACTACGTCCTTTGACAGCCG
QPKC1F	GGTGTGCTTGGCGTTGAAAT
QPKC1R	AGGCGATTGTTGGAGAAGCA
QBCK1F	ACCGCAAGATCCAAGAAGGG
QBCK1R	CGCGAAAGATCGTCTACGGA
QMKK1F	GCCACACCGATGGGAAGTAA
QMKK1R	CTGGTGTTCCTTGCGGTTTG
QSlt2F	TCGCAGAATTACTTGGCGGT
QSlt2R	AAAGGTCGCTTGGCCATGTA

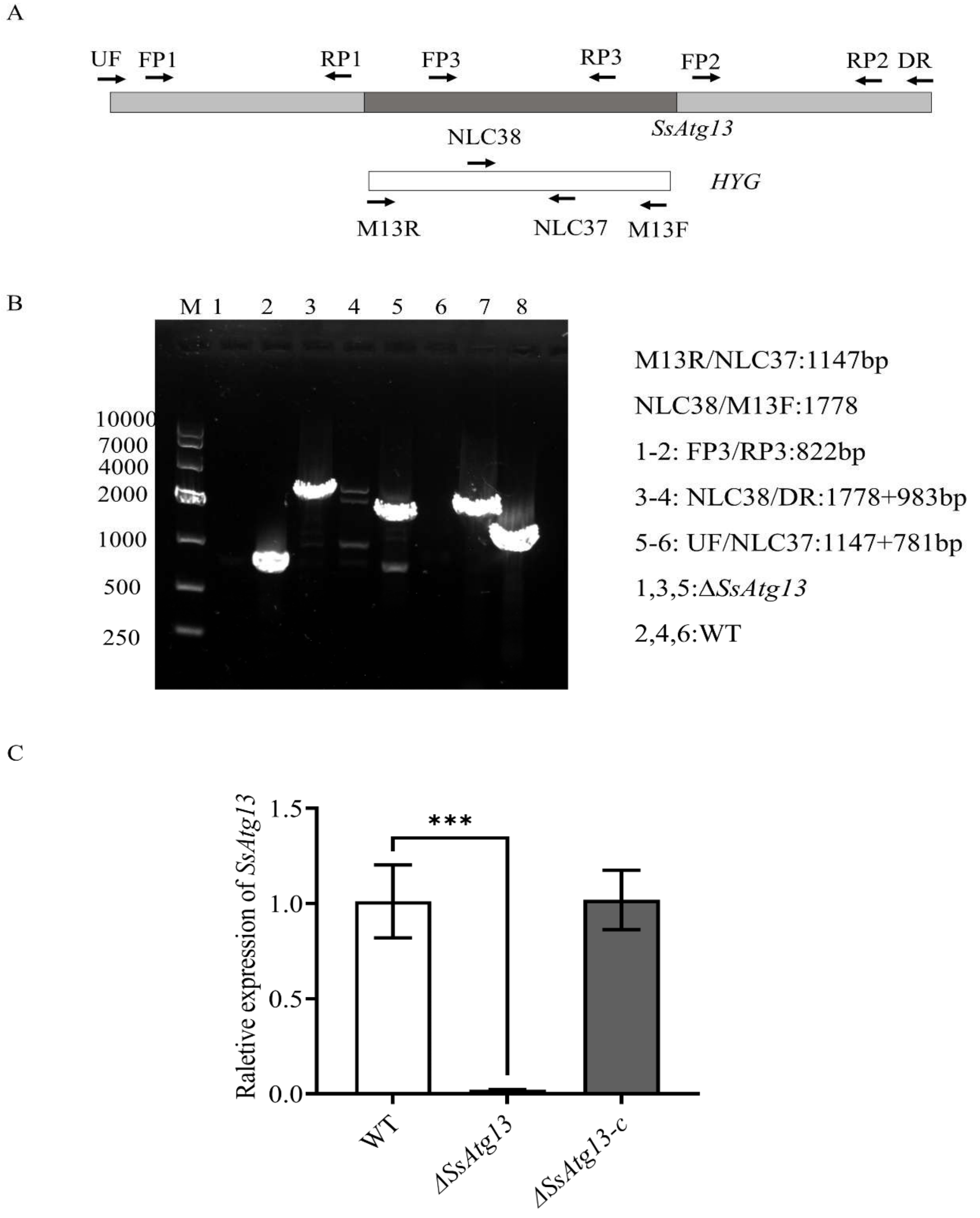

Fig. S1 SsAtg13 knockout and complement verification

(a) Gene knockout method; (b) PCR verification of target gene knockout and hygromycin gene insertion; (c) The gene expression level of the transformed suborder was verified by QPCR.

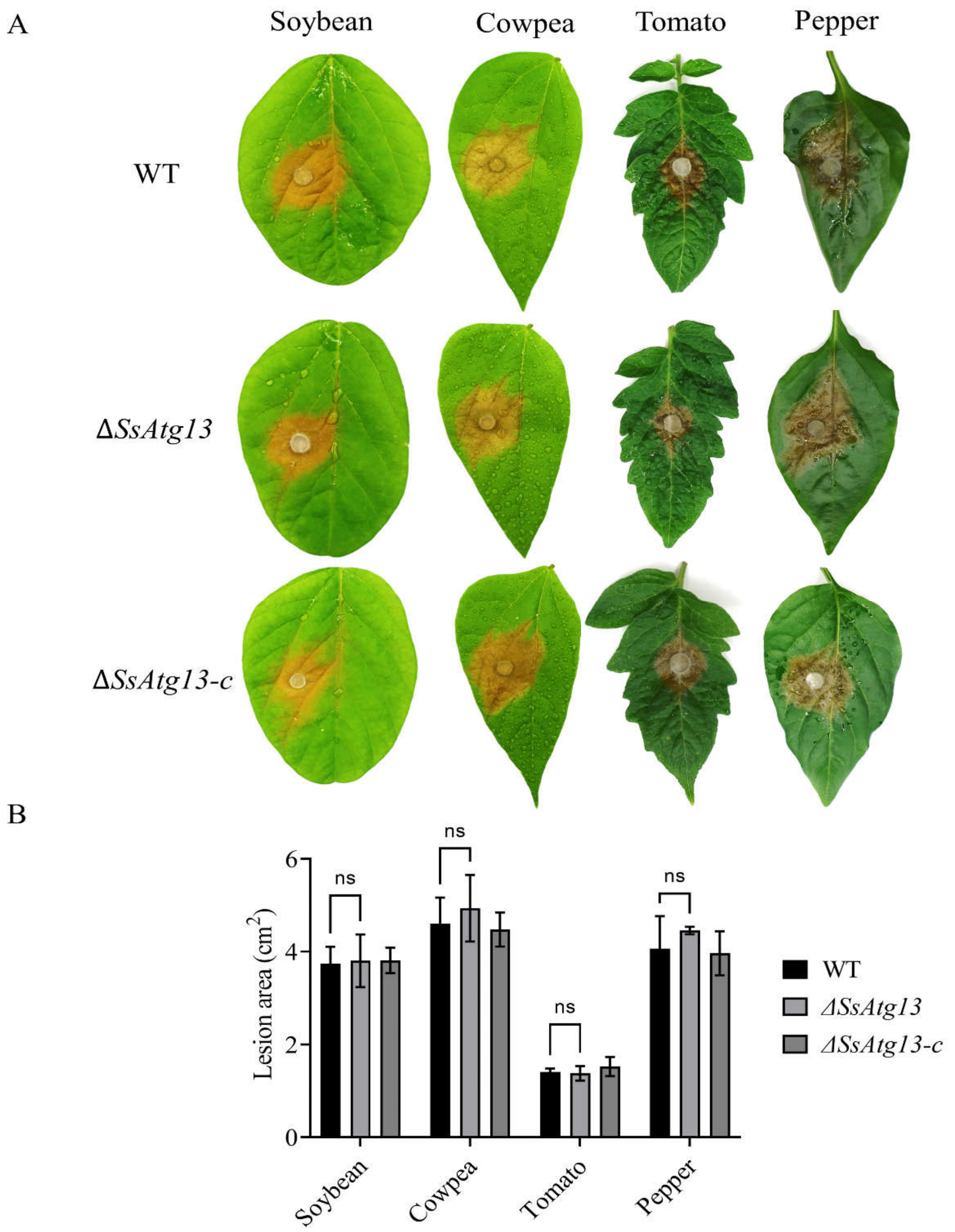

Fig. S2 SsAtg13 deletion did not affect the pathogenicity of *S. sclerotiorum*

（a）Pathogenicity analysis of Δ*SsAtg13*; (b) Determination of lesion diameter after Δ*SsAtg13* inoculation

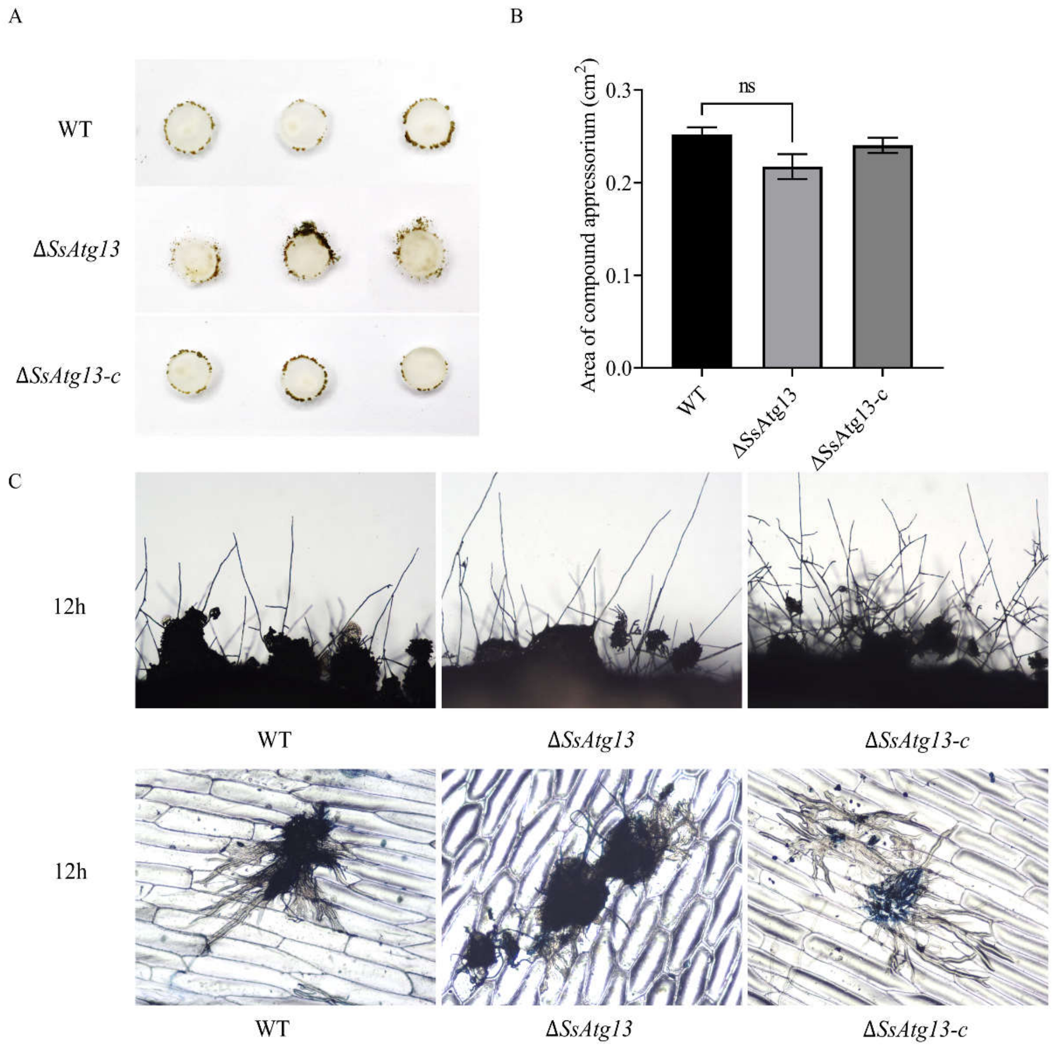

Fig. S3 Δ*SsAtg13* did not affect compound appressoria formation and penetration.

(a,b) Compound appressoria of strains on hydrophobic interface. Image J was used to analysis of compound appressoria (c) Compound appressoria formation on onion epidermal cells.
